# Preparation of hybrid samples for scanning electron microscopy (SEM) coupled to focused ion beam (FIB) analysis: A new way to study cell adhesion to titanium implant surfaces

**DOI:** 10.1371/journal.pone.0272486

**Published:** 2022-08-02

**Authors:** Ludovica Parisi, Andrea Toffoli, Benedetta Ghezzi, Paola Lagonegro, Giovanna Trevisi, Guido M. Macaluso

**Affiliations:** 1 Department of Orthodontics and Dentofacial Orthopedics, Laboratory for Oral Molecular Biology, University of Bern, Bern, Switzerland; 2 Centro Universitario di Odontoiatria, Università di Parma, Parma, Italy; 3 Dipartimento di Medicina e Chirurgia, Università di Parma, Parma, Italy; 4 CNR-SCITEC, Milano, Italy; 5 IMEM-CNR, Parma, Italy; William & Mary, UNITED STATES

## Abstract

The study of the intimate connection occurring at the interface between cells and titanium implant surfaces is a major challenge for dental materials scientists. Indeed, several imaging techniques have been developed and optimized in the last decades, but an optimal method has not been described yet. The combination of the scanning electron microscopy (SEM) with a focused ion beam (FIB), represents a pioneering and interesting tool to allow the investigation of the relationship occurring at the interface between cells and biomaterials, including titanium. However, major caveats concerning the nature of the biological structures, which are not conductive materials, and the physico-chemical properties of titanium (*i*.*e*. color, surface topography), require a fine and accurate preparation of the sample before its imaging. Hence, the aim of the present work is to provide a suitable protocol for cell-titanium sample preparation before imaging by SEM-FIB. The concepts presented in this paper are also transferrable to other fields of biomaterials research.

## 1. Introduction

The interaction among tissue components and titanium surfaces is crucial in order to ensure a successful clinical outcome in implant dentistry. More in details, implants stability is determined by the intimate structural and functional connection, which occurs at the interface between titanium and the alveolar bone, namely osseointegration [1–3]. Furthermore, in order to ensure a successful clinical outcome, tight soft tissue attachment around implant abutment is also regarded as important as osseointegration. Indeed, the formation of a proper seal around implant neck avoids complications such as biofilm formation with consequent peri-implant tissue inflammation and related issues [4, 5].

At a cellular level, both the osseointegration and the seal formation are controlled by the firm attachment, adhesion and spreading of the local tissue-resident cells to the titanium surface. Therefore, detailed *in vitro* study of the interactions occurring between these cellular components and titanium could be informative and provide important insights into the mechanisms behind the success of these clinical options.

Over the last decades, numerous microscopy techniques have been proposed to study the adhesion and the shape of cells on different type of biomaterials, including titanium [6–8]. However, to the best of our knowledge, no clear winning method has been described to qualitatively and quantitatively study the interaction among cells and the underlying surface. Optical techniques, such as fluorescence interface contrast microscopy (FLIC), reflection interference contrast microscopy (RICM) or total internal reflection fluorescence microscopy (TIRF) require the use of transparent substrates for the study of cell-surface junction, which is obviously not applicable to titanium [9–12]. While, on the other hand, also high-resolution and powerful techniques, such as transmission electron microscopy (TEM), present some limitations: they require the sectioning of the samples in ultra-thin slices (<100nm), which are not suitable for volumetric reconstructions [13, 14]. Additionally, all the current methods of imaging present limitations in studying the interaction of cells with materials that present high structured and complex surface topography, adding further challenge to the study of the interactions occurring between cells and intricated structures, such as microrough implant surfaces.

Among electron microscopy techniques, scanning electron microscopy (SEM) has long been recognized as the most viable option to image and study the three-dimensional (3D) structure of objects, with no arguably instrument with its breadth of applications. Its function is based on the use of an accelerated electron beam, with a wavelength 100000 shorter than that of light photons, which makes possible to enhance the magnification power of light microscopies (200nm) to 1000-fold (0.2nm). In details, SEM works thorugh an high energy electron beam, which scans across the surface of a conductive specimen, thus inducing the emission of other electrons (secondary), which are collected, processed and converted into 2D images. Over the past years, SEM has been improved with numerous investigation tools. Recently, SEM coupling with an additional column capable to generate a focused ion beam (FIB) for the live milling or cross-sectioning of samples at a glancing defined angle has been proposed [15, 16]. Accordingly, we hypothesized to use SEM-FIB microscopy as a viable tool to study the interaction occurring at the cell-titanium interface [17]. However, the analysis of cellular and biological structures at these high resolution has always been a challenge, because of their intrinsic non-conductive nature.

Based on these premises, this protocol was developed specifically to prepare cell-titanium specimen before SEM-FIB analysis, in order to determine and study what happens at their interface. Additionally, we would like to aknowledge that the concepts presented here are further transferrable to the study of other types of biomaterials.

## 2. Materials and methods

The protocol described in this peer-reviewed article is published on protocols.io, at the following doi number dx.doi.org/10.17504/protocols.io.36wgq42kyvk5/v1. Additionally, the described protocol is also included for printing as S1 File with this article.

### 2.1 Titanium discs

Commercially pure, grade 4 (ISO5832/2) titanium discs with sandblasted/acid etched surface were kindly provided by Sweden&Martina (Due Carrare, Padova, Italy). Samples were provided as sterile discs of 3.5mm (thickness) and 8.0 mm (diameter) to fit in a 48-well plate. In order to increase the hydrophilicity of the specimens and to obtain the hydrophilic-Ti sample, part of the discs was thermally treated at 300°C for 2h in a programmable oven (Programat P60, Ivoclar Vivadent, Schann, Lichtenstein).

### 2.2 Titanium discs characterization

To observe any difference in the surface microtopography between Ti and hydrophilic-Ti discs, samples were analyzed by SEM, using a dual beam Zeiss Auriga Compact system equipped with a GEMINI Field-Effect SEM column (Zeiss, Oberkochen, Germany). The analysis was performed at 5keV. Furthermore, surface hydrophilicity was assessed by measuring the contact angles between the surface and 5μl water droplet using 10 titanium surfaces per group.

### 2.3 Cell culture

Human osteosarcoma-derived MG-63 were obtained from the American Type Culture Collection (LGC, Standards s.r.l., Sesto S. Giovanni, MI, Italy). Cells were cultured in Dulbecco’s Modified Eagle Medium (DMEM, Thermo Fisher Scientific, Waltham, MA, USA), 1% L-Glutamine (Thermo Fisher Scientific) and 1% Penicillin and Streptomycin (PenStrep, Thermo Fischer Scientific). Upon confluence cells were trypsinized and seeded on titanium specimen at a final concentration of 10000cells/disc.

### 2.4 Immunofluorescence

24h after seeding, cells were fixed in 4% paraformaldehyde (PFA, Sigma-Aldrich, Saint-Louis, CA, USA) for 20min at room temperature (RT), rinsed 3 times in PBS, permeabilized with 0.1%v/v Triton-X-100 (Sigma-Aldrich) at RT, blocked in BSA 1%w/v for 30min at RT and incubated with a primary mouse anti-vinculin antibody (Clone 7F9, FAK100, Sigma-Aldrich) for 1h at RT. Cultures were washed 3 more times with PBS, incubated with a secondary anti-mouse fluorescent FITC-labeled secondary antibody (Molecular Probes, Thermo Fisher Scientific) and TRITC-conjugated phalloidin for 1h at RT protected from the light, washed 3 more times in PBS, incubated with DAPI (FAK100, Sigma-Aldrich) for 5min at RT and finally mounted under glass cover slips using the anti-fade-mounting medium (P7481, Thermo Fisher Scientific) for photo bleaching reduction.

All the samples were analyzed under an equipped for fluorescence Zeiss Axio Imager A.2 (Carl Zeiss, Jena, Germany).

### 2.5 SEM-FIB

24h after seeding, cells were washed in PBS and fixed in 2.5%w/v glutaraldehyde in 0.1M sodium cacodylate buffer for 30min at RT and rinsed in 0.1M sodium cacodylate buffer for 5min at RT. Subsequently, samples were dehydrated in ethanol (EtOH) at increasing concentrations (35%, 50%, 70%, 95% and 99%). Each EtOH was maintained for 10min at RT. Therefore, samples were critical point dried with liquid carbon dioxide (CPD 030 Baltec, BALTEC, Wallruf, Germany) and covered by a nm thick gold layer (PLANO, Wetzlar, Germany) using a SCD 040 Coating device (Balzer Union, Wallruf, Germany).

Specimens were analyzed using a dual beam Zeiss Auriga Compact system equipped with a GEMINI Field-Effect SEM column and a Gallium FIB source (Carl Zeiss). SEM analysis was performed at 5keV, while the Gallium ion beam for the cross-sectional analysis was accelerated at 30kV with a 500pA current. SEM-FIB images were analyzed while acquiring. Cell-titanium distance and cell thickness were indeed investigated for 10 cells on each samples with the designated tool of the interface software.

### 2.6 Statistical analysis

Data were analyzed using Prism7 (GraphPad, La Jolla, CA, USA) and are reported as the mean±SD. Differences between groups were evaluated with t-test and considered significant when p<0.05.

All the experiments were performed three times in multiple replicates.

## 3. Expected results

To assess the potential of the described protocol for the preparation of the cell-titanium samples before SEM-FIB imaging, we would like to report a practical example.

In the latest 20 years, major achievements in dental implants amelioration have been obtained by targeting implant surface characteristics. Most importantly, chemical modifications to increase the hydrophilicity of microrough surfaces have been shown to drastically optimize the osseointegration of implantable materials by increasing the bone-to-implant contact (BIC) and accelerating new osteogenesis [18, 19]. However, in spite these modifications have been successfully employed in preclinical [20, 21] and clinical studies [22–24], the way the bone cells adhere when in contact with these surfaces is still largely unexplored. Exploiting the protocol described in this article, we were the firsts able to show how bone-like cells interact with titanium surfaces during adhesion [17, 25, 26].

**Fig 1:**
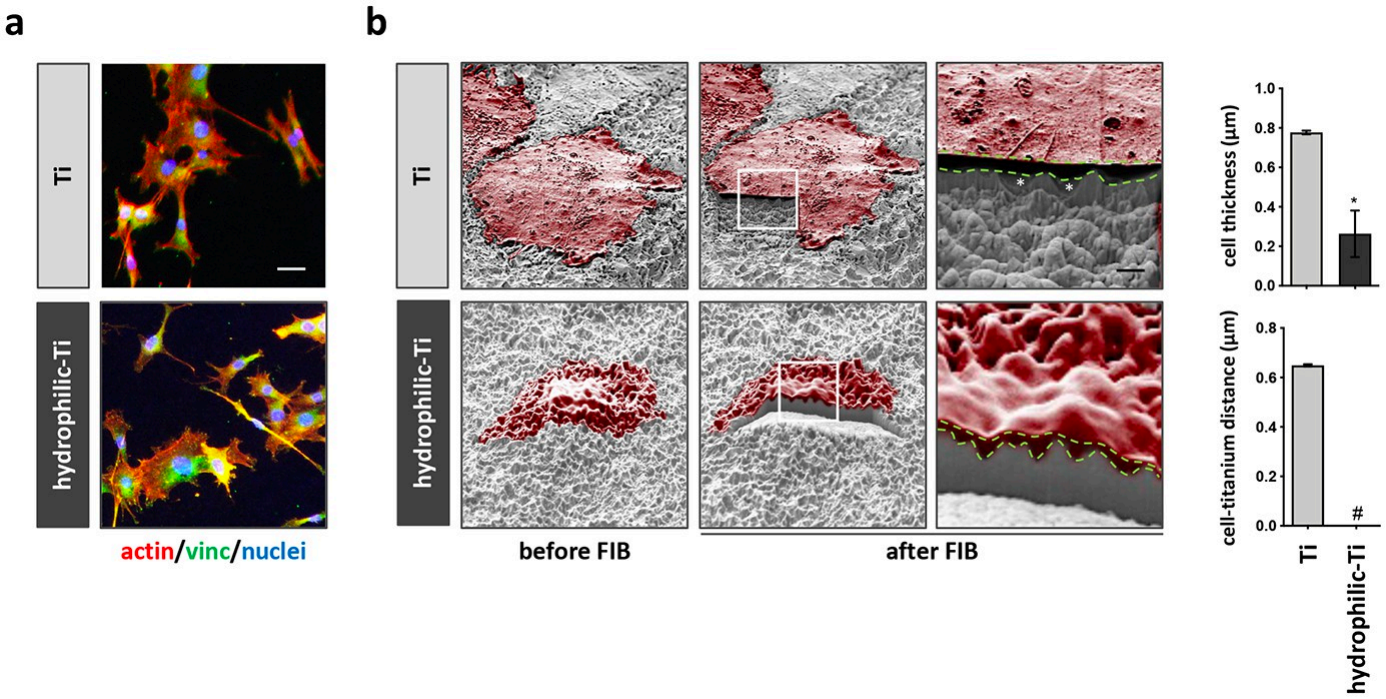
Interface intimate relationship between human MG-63 and microrough titanium implant surfaces with normal or enhanced hydrophilicity. Human MG-63 were cultured on microrough titanium implant surface with standard (Ti) or enhanced hydrophilicity (hydrophilic Ti), thus prepared and analyzed by fluorescence or by SEM-FIB microscopy. (**a**) Immunofluorescence staining for actin (red), vinculin (green) and cell nuclei (blue) reveals the polygonal shape of human MG-63 when cultured on titanium surfaces. Scale bar: 50μm. (**b**) SEM images before and after FIB cross-sectioning reveals the shape and the intimate connection established by the cells (red) with the underlying surface by human MG-63. Scale bar: 2μm. Scale bar close up: 0.2μm. The intimate relationship between cells and titanium was further quantified by measuring cell thickness and the distance occurring between the cell border and titanium. * = p<0.05. Green dashed lines indicate the top and bottom border of the cells, while white stars indicate air bubbles entrapped between cells and titanium.

In Fig 1 the look of human MG-63 cultured on a microrough (Ti) or on hydrophilic microrough titanium (hydrophilic-Ti) surface is presented (see surface characterization is reported in S1 Fig and [27]). To highlight the potential of SEM-FIB microscopy in adding qualitative and quantitative information to our analysis, we compared the images obtained by SEM-FIB investigation, to the images obtained by fluorescence microscopy, which is a largely used technique to visualize cells on titanium.

By fluorescence microscopy, the informations connected to the adhesion of human MG-63 to different titanium surfaces are limited to the shape the cells. On both the tested surfaces, human MG-63 present with a typical healthy spindle-like shaped morphology (Fig 1a). Although with IF analysis, it is possible to quantify the number of focal adhesion expressed by the cells on the two surfaces, which may correlate with the strength of cell adhesion to the underlying surface [25], no clear conclusion on the way the cells interact with surface itself can be properly drawn. On the other hand, SEM-FIB analysis produced significant more informations regarding the different type of adhesion of human MG-63 to Ti or to hydrophilic-Ti surfaces (Fig 1b). SEM analysis shows that hydrophilicity promotes a closer adhesion of the cells to the surface, further allowing to glimpse the underlying micro-texture of titanium. Conversely, this is not distinguishable under the cell soma of cells cultured on the canonical Ti surface. However, with the SEM observation only, a more accurate investigation, of the interactions that occur underneath the cell body is not possible. Amazingly, after FIB cross-sectioning of the cells, it is evident that the standard Ti surface is prone to the entrapment of air bubbles that interfere with the tight and close adhesion of the cells to the surface (Fig 1b *close up yellow star*). As a consequence, cells remained suspended and did not properly flatten on the Ti sample. Additionally, even though the SEM analysis hardly allowed to distinguish the layout of cells on the hydrophilic-Ti, the FIB cross-sectional analysis evidences the prominence of the biological material. Noteworthy, no air bubbles were found to be entrapped under the cell soma in this case. Furthermore, by analysing the FIB-cut cross-section of the sample, it has been possible to measure the cleft distance in between cell membrane and titanium, as well as the thickness of the cells. In both the cases, significant differences have been found between the two different surfaces (cell-titanium distance p = 0.0017; cell thickness p<0.0001). To the best of our knowledge, it is not yet clear whether a closer adhesion of the cells to the titanium surface is responsible for the improved outcomes derived from the use of more hydrophilic surfaces. However, former studies have shown that a pre-coating of titanium surfaces with fibronectin, a well-known protein involved in cell adhesion, improve cell adhesion in similar ways and fashion [26]. Hence, we can speculate that increased hydrophilicity of titanium implants may cause a better adhesion of the cells generating similar effects to the one produced by fibronectin pre-adsorption. Moreover, we previously showed that when cultured on more hydrophilic titanium surfaces, murine MC3T3-E1 osteoblasts expressed more focal contact points, which is known to be putative for a better cell adhesion [28]. Here, we add further evidence to this observation, and we can further speculate that a higher number of focal adhesion, homogeneously distributed in the whole cell can be correlated to the closer adhesion we could observed by SEM-FIB analysis. Clearly, a more depth investigation could be performed by using an immunogold labelling approach for vinculin. However, we must admit we did not yet optimized a protocol for our purposes.

In conclusion, these images provide clear evidence of the potential of the proposed investigation protocol, and further provide an unprecedent possibility to investigate the spatial and geometric relationship among cells and biomaterials.

## Supporting information

S1 File Protocol for SEM-FIB preparation from protocols.io. (DOCX)

S1 Fig Surface characterization of titanium implant surfaces with normal or enhanced hydrophilicity. (TIF)
