# Supplementary material for: Preparation of hybrid samples for scanning electron microscopy (SEM) coupled to focused ion beam (FIB) analysis: A new way to study cell adhesion to titanium implant surfaces
Source: PLoS One. 2022 Aug 2;17(8):e0272486. doi: 10.1371/journal.pone.0272486 (PMC9345346; doi:10.1371/journal.pone.0272486)
Supplement: S1 File — (DOCX) [file pone.0272486.s001.docx]

**S1 File: Preparative for SEM-FIB analysis protocol**

We report the complete sample preparation protocol for SEM-FIB imaging of a single cell layer. The timings suggested for each step of the protocol should be adapted if other types of biological samples (i.e. tissues) are used.

**S1.1 Fixative**

The first step to obtain high quality images concerns fixative, namely the immobilization of the structures on the material surface. Fixative is generally critical because it requires the use of chemical agents, that may penetrate the cellular and biological structures, thus damaging their overall make-up.

**Reagents needed:**

- **PBS:** Phosphate Buffer Saline
- **Na-Cacodylate buffer:** Na-Cacodylate buffer 0.1M
- **Glutaraldehyde:** Glutaraldehyde 2.5% w/v in Na-Cacodylate buffer 0.1M

**Step for fixative:**

1. Remove culturing medium from the samples;
2. Rinse samples twice with PBS;
3. Cover samples with Glutaraldehyde;
4. Incubate samples for 30 minutes at room temperature;
5. Remove Glutaraldehyde from the samples;
6. Cover samples with Na-Cacodylate buffer;
7. Incubate samples for 5 minutes at room temperature;
8. Remove Na-Cacodylate buffer.

**S1.2 De-hydration**

After the fixation, attention must be put on sample de-hydration, which allows to remove the water content in the specimen. This step is necessary in order to obtain high quality SEM images.

**Reagents needed:**

- **EtOH 35%:** Ethanol 35% v/v in ddH_2_O
- **EtOH 50%:** Ethanol 50% v/v in ddH_2_O
- **EtOH 70%:** Ethanol 70% v/v in ddH_2_O
- **EtOH 95%:** Ethanol 95% v/v in ddH_2_O
- **EtOH 99%:** Ethanol 99% v/v in ddH_2_O

**Step for fixative:**

1. Cover samples with EtOH 35%;
2. Incubate samples for 10 minutes at room temperature;
3. Remove EtOH 35% and cover samples with EtOH 50%;
4. Incubate samples for 10 minutes at room temperature;
5. Remove EtOH 50% and cover samples with EtOH 70%;
6. Incubate samples for 10 minutes at room temperature;

(NB! At this time point protocol could be interrupted and samples stored in EtOH 70% at 4°C)

1. Remove EtOH 70% and cover samples with EtOH 95%;
2. Incubate samples for 10 minutes at room temperature;
3. Remove EtOH 95% and cover samples with EtOH 99%;
4. Incubate samples for 10 minutes at room temperature.

**S1.3 Sputter coating**

Sputter coating with a thin layer of conductive material is mandatory for SEM imaging of biological components, due to their non-conductive nature. This feature leads to an undesired accumulation of electrons on the sample surface, resulting in images with well-known artifacts, such as extended extra-white regions. As a sputter coating material, we suggest the use of Gold (Au). Indeed, Au combines high conductivity to a relatively small size of the ion. However, also other coating materials such as platinum (Pt), palladium (Pd) or silver (Ag) can be used used. The optimal setting of coating parameters is important, and particular attention should be provided to the time of sample exposure to the coating material. Artifacts like the effect of a plentiful snowfall, with consequent alteration of material surface topography and hiding of specimens’ features, as well as the generation of white regions because of electrons accumulation in the sample should be avoided.

**Step for sputter coating:**

1. Put the samples into a sputtering machine and drive it to the vacuum;
2. Reach the critical point dry of the samples with carbon dioxide (CO_2_);
3. Sputter the samples with gold for 90s.
