# Supplementary figures and images for: Preparation of hybrid samples for scanning electron microscopy (SEM) coupled to focused ion beam (FIB) analysis: A new way to study cell adhesion to titanium implant surfaces
Source: PLoS One. 2022 Aug 2;17(8):e0272486. doi: 10.1371/journal.pone.0272486 (PMC9345346; doi:10.1371/journal.pone.0272486)

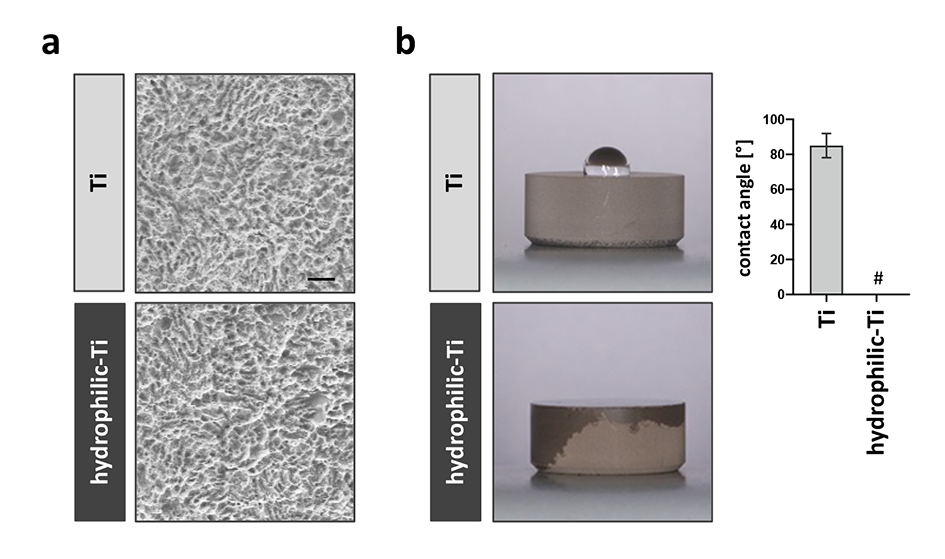

Supplement: S1 Fig — (TIF) [file pone.0272486.s002.tif]
